# Alterations in intestinal development and barrier function in response to dietary β-mannanase in growing broilers

**DOI:** 10.3389/fvets.2026.1751066

**Published:** 2026-06-08

**Authors:** Xiaofei Yang, Yuying Huang, Pingyu Xia, Rui Zheng, Guangming Zhang, Qiong Liu, Jian Li, Xuejun Li, Wen Chen, Huaiyong Zhang

**Affiliations:** 1 Key Laboratory of Animal Biochemistry and Nutrition, College of Animal Science and Technology, Ministry of Agriculture, Henan Agricultural University, Zhengzhou, China; 2 Beijing Strowin Biotechnology Co., Ltd., Beijing, China; 3 Key Laboratory for Animal Disease-Resistance Nutrition of China, Institute of Animal Nutrition, Ministry of Education, Sichuan Agricultural University, Ya’an, Sichuan, China

**Keywords:** barrier function, growing broilers, growth performance, intestine development, β-mannanase

## Abstract

This study evaluated the effects of dietary β-mannanase supplementation on growth performance, intestinal development, nutrient digestibility, and cecal microbiota in growing broilers. A total of 160 chicks were assigned to one of two dietary treatments (8 replicates of 10 birds each) and were fed a basal corn–soybean meal diet (Ctrl) or a basal diet supplemented with 150 mg/kg β-mannanase until day 21. With similar body weight, β-mannanase supplementation significantly reduced feed intake and improved the feed conversion ratio from day 1 to 21 (*p* < 0.05). Compared with the Ctrl group, β-mannanase supplementation reduced chyme viscosity and upregulated jejunal mRNA expression of sodium-dependent glucose cotransporter 1 (*SGLT1*) and fatty acid-binding protein 6 (*FABP6*) (*p* < 0.05). Although the addition of β-mannanase to the diet did not affect the length and weight of the duodenum and jejunum, it notably increased the villus height-to-crypt depth ratio in the jejunum (*p* < 0.05), as well as the DNA content, a quantitative parameter of enterocytes, in both the duodenum and jejunum, contributing to a significant increase in the digestibility of dry matter and metabolizable energy. Regarding barrier integrity, β-mannanase supplementation decreased serum D-lactic acid and endotoxin levels and upregulated jejunal expression of zonula occludens-1 (*ZO-1*), *occludin*, and *mucin-2* at day 21 (*p* < 0.05). Furthermore, β-mannanase supplementation significantly increased the levels of secretory immunoglobulin A (sIgA) and reduced the expression of interleukin-1 beta (IL-1β) in the jejunum. Cecal microbiota analysis revealed that β-mannanase increased the relative abundance of *g_Alistipes_A_871400*, *g_Lachnoclostridium_A_130679*, *g_Enterocloster*, and *g_Comamonas_F_58925*. These findings indicate that dietary β-mannanase improves gut micro-ecology by reducing chyme viscosity, enhancing intestinal barrier function, and modulating microbiota composition, thereby promoting nutrient utilization and feed efficiency in growing broilers.

## 1 Introduction

Although corn and soybean meal are well established as the primary feedstuffs in poultry diets due to their high nutritional value, raw material shortages and changing international circumstances have led to the increasing use of more economical by-products, such as cottonseed meal and corn gluten meal, despite their lower nutritional quality (1, 2). However, a significant portion of these feed ingredients contains non-starch polysaccharides (NSP), including cellulose, xylan (and arabinoxylan), and β-mannans (and β-galactomannan) (3). β-mannan, a soluble NSP composed of repeating mannose units connected by β-1,4-glycosidic bonds, is present in a wide variety of commonly used feed ingredients. Soybean meal is the most representative source, typically containing approximately 1–3% β-mannan (4). These β-mannans act as anti-nutritional factors that are highly resistant to digestive enzymes and increase intestinal chyme viscosity, consequently impairing nutrient absorption in the intestinal mucosa in broilers (5). Furthermore, since mannans of various configurations are present on the surface of numerous pathogens, including fungi, bacteria, and viruses, β-mannan in diets is believed to induce adverse physiological effects associated with immune activation and intestinal inflammation (3, 4, 6). β-mannanase is one of the endo-carbohydrases that hydrolyzes β-mannans in feed into mannan-oligosaccharides (MOS) and mannose (7). Supplementing broiler diets with β-mannanase has been shown to enhance growth performance and partially counteract these anti-nutritional effects in the digestive process by reducing digesta viscosity and improving nutrient digestibility (6, 8, 9). Additionally, MOS are recognized as prebiotics that positively modulate immune responses and gut microbial composition in broilers (10). Accordingly, β-mannanase supplementation helps optimize the intestinal environment, as shown by improved intestinal morphology, reduced inflammation, and increased abundance of beneficial bacteria—changes potentially mediated indirectly through MOS (11, 12).

Early maintenance of intestinal health is critical in broilers, as the immaturity of intestinal function and immune capacity often leads to impaired nutrient utilization and weakened defensive systems, particularly when birds are exposed to environmental and dietary risk factors (13, 14). Consequently, poultry nutritionists have shown growing interest in using exogenous enzymes such as β-mannanase to support intestinal health (15). However, the efficacy of β-mannanase supplementation varies considerably, depending on animal species, physiological stage, diet composition, and even the enzyme inclusion level (6, 16, 17). Although numerous studies have documented its positive effects on the performance, nutrient utilization, and host health of poultry (broilers and laying hens) (3, 6), the results in broilers remain inconsistent. Moreover, limited information is available about how β-mannanase influences comprehensive intestinal health and gut microbial composition during the early growth stage of broilers.

Given this background, the present study was conducted to investigate the effects of dietary β-mannanase supplementation on growth performance, intestinal development, nutrient digestibility, and cecal microbiota in growing broilers. Furthermore, we hypothesized that adding commercial β-mannanase to a corn–soybean meal-based diet would enhance the growth performance of growing broilers by promoting intestinal development, improving intestinal barrier function, and increasing nutrient digestibility.

## 2 Materials and methods

### 2.1 Ethical statement

All procedures involving broiler chickens used in this study were approved by the Animal Care and Use Committee of Henan Agricultural University (approval no. HAU 2024-03) and were conducted in accordance with the guidelines for animal welfare and experimental protocols.

### 2.2 Animals, diets, and experimental design

The broiler chicks, obtained from a local hatchery, were allowed *ad libitum* access to both feed and water. The temperature during the experiment was lowered gradually from 30 to 32 °C at 1–3 days of age and then further reduced to 21 °C by 21 days of age. The light schedule was 23 L:1D during days 1–7 and 18 L:6D thereafter. Birds were vaccinated against Newcastle Disease and Infectious Bronchitis on day 1 of age. Using a randomized block design, a total of 160 1-day-old AA broilers were allocated into the Ctrl group (fed with a basal diet) and the β-mannanase group (basal diet supplemented with 150 mg/kg β-mannanase) based on body weight, with each treatment including 8 replicates of 10 birds each. The experimental period lasted from day 1 to day 21 of age. The basal diet was formulated to meet or exceed the nutritional requirements of the broilers, and the composition of the experimental diet is shown in Table 1. β-mannanase (Shengmei Enzyme, activity 3,000 U/g) was provided by Beijing Strowin Biotechnology Co., Ltd. (Beijing, China). All treatment diets were prepared in pelleted form.

**Table 1:** Composition and nutrition level of the experimental diets (1–21 days, as-fed basis).

Items	Ingredients, %	Items	Nutrient composition, %
Corn	43.72	Crude protein	22
Flour	16. 00	Apparent metabolic energy	29.50
Soybean oil	2.30	Dry matter	86.52
Soybean meal	26.3	Ether extract	4.72
Cottonseed meal	3.0	Crude fiber	2.69
Corn gluten meal	4.5	Crude ash	5.93
Sodium chloride	0.25	Calcium	0.88
Limestone	1.05	Total phosphorus	0.68
Montmorillonite	0.20	Avail phosphorus	0.46
CaHPO_4_	1.66	Digestibility lysine	1.30
Choline chloride	0.10	Digestibility methionine	0.56
L-lysine HCL	0.83	Digestibility tryptophan	0.20
DL-methionine	0.25		
L-threonine	0.24		
Mineral premix^a^	0.20		
Vitamin premix^b^	0.30		
Total	100.0		

a Provided per kilogram of diet: Cu (CuSO_4_∙5H_2_O), 8 mg; Fe (FeSO_4_∙7H_2_O), 80 mg; Zn (ZnSO_4_∙7H_2_O), 90 mg; Mn (MnSO_4_∙H_2_O), 70 mg; Se (NaSeO_3_), 0.3 mg; I (KI), 0.4 mg.

b Provided per kilogram of diet: retinol, 2.06 mg; cholecalciferol, 0.04 mg; vitamin E, 30.01 mg; thiamine, 1 mg; riboflavin, 3.9 mg; pyridoxine, 3.375 mg; vitamin B12, 0.01 mg; calcium pantothenate, 8.85 mg; folate, 0.5 mg; biotin, 0.1 mg; niacin, 49.25 mg.

### 2.3 Sample collection and measurement

At 21 days of age, chicks were weighed after an 8 h feed withdrawal period. The body weight and feed intake were recorded, and weight gain and feed conversion ratio (feed-to-weight gain ratio) were calculated. One bird from each replicate was selected for sampling based on the average body weight of each pen. Blood was collected via the jugular vein, then centrifuged at 4,000 × *g* for 10 min at 4 °C to obtain serum, and stored at −80 °C until analysis. After euthanasia, all chicks were slaughtered, and the full duodenum and jejunum sections were taken for the measurement of weight and length. The midpoint of two intestinal segments was cut into pieces within 1 cm and rinsed gently with phosphate-buffered saline (PBS); then, these tissues were fixed immediately in 4% (w/v) paraformaldehyde solution for histological examination. Subsequently, the digestive tract was removed to collect digesta samples from the jejunum and cecum, as well as mucosa samples from the duodenum and jejunum. Jejunal and cecal digesta samples were then placed in either 15-mL tubes on ice for viscosity analysis or stored at −80 °C for gut microbial analysis. Mucosal samples were collected by scraping the intestinal mucosa with a glass slide and snap-frozen in liquid nitrogen and stored at −80 °C for later analysis.

### 2.4 Determination of apparent digestibility

A 7-day metabolizable trial was conducted from day 17 to 23, consisting of a 4-day acclimatization period followed by a 3-day excreta collection period. The broilers were randomly allocated to 1 of 16 metabolism cages. Birds subjected to 48 h of feed withdrawal received the basal and β-mannanase diets (*n* = 8/test diet) by orogastric administration based on 2% of live body weight, respectively. Excreta samples were collected three times daily using their respective collection plastic bags and stored at −20 °C until analysis. The samples were weighed and then stored −20 °C immediately. These fecal and feed samples were dried at 65 ± 5 °C for 24 h, weighed, and crushed to pass through a 40-mesh sieve for assessments of dry matter (DM), crude protein (CP), and gross energy (GE). Dry matter and nitrogen (N) were determined according to Association of Official Analytical Chemists (AOAC, 2006), while gross energy was analyzed using a Parr oxygen bomb calorimeter (6200 Isoperibol Calorimeter, Parr Instrument Company, Moline, IL, USA) with benzoic acid as the calibration standard. In addition, since growing birds retain nitrogen for muscle growth, the apparent metabolizable energy (AME) is often corrected to zero N retention, which is called the N-corrected AME (AMEn), to provide a more accurate energy value by removing the energy cost of N excretion. For this study, AMEn was calculated using the method described by a previous study (18), as shown below:

$$N\;\text{retention}\;(g)=29\;g/\mathrm{kg}\times \frac{\text{Body weigh gain}\;(g)}{1000};$$

$$\text{AMEn of basal diet}=\frac{\left(\mathrm{GE}\;\text{intake}-\mathrm{GE}\;\text{excretion})-(8.22\times N\;\text{retention}\right)}{\mathrm{DM}\;\text{intake}}$$

### 2.5 Intestinal permeability and immune status

The concentrations of diamine oxidase (DAO) and D-lactic acid (D-LA) in serum, as well as secretory immunoglobulin A (sIgA) in both the duodenum and jejunum, were determined using commercially available enzyme-linked immunosorbent assay (ELISA) kits (MEIMIAN Biotechnology Co., Ltd., Jiangsu, China). Endotoxin content in serum was determined using the Chromogenic End-point Tachypleus Amebocyte Lysate (CE TAL) kit, according to the manufacturer’s protocol (Nanjing Jiancheng Bio-Engineering Institute).

### 2.6 Viscosity measurement

Following the procedure by Duarte et al. (19), samples of jejunum digesta were centrifuged at 1,000 × *g* at 4 °C for 10 min, and the obtained supernatant was further transferred to a 2-mL tube and centrifuged at 10,000 × *g* at 4 °C for 10 min. Ultimately, 0.5 mL of the supernatant was placed in the viscometer set at 25 °C to measure viscosity (Brookfield Digital Viscometer, Model DV-II Version 2.0, Brookfield Engineering Laboratories Inc., Stoughton, MA, USA). The result was calculated as the average viscosity at 45.0 and 22.5 per s shear rates, and the value was recorded in centipoise (cP = 1/100 dyne s/cm^2^).

### 2.7 Intestinal morphology

The intestinal fragments were sectioned according to standard histological techniques, dehydrated, and embedded in paraffin. Then, staining was performed using hematoxylin and eosin (H&E) staining for the measurement of villus height and crypt depth. In addition, paraffin-embedded fixed intestinal tissues were stained with Alcian blue–Periodic acid–Schiff (AB-PAS) to determine the mucous layer. Villus height, crypt depth, and mucous layer were measured using a digital camera attached to an Olympus CX31 microscope (Lumenera Corporation, Ottawa, Canada), and the villus height-to-crypt depth ratio was subsequently calculated. The lengths of 10 well-oriented intact villi and their associated crypts were assessed in each slide, and the average result of 10 measurements per sample was reported as a single value per bird.

### 2.8 Biochemical indices of intestinal mucosal growth

DNA changes reflect variations in cell population, as the DNA content of diploid cells remains unchanged following cell formation. The protein-to-DNA ratio serves as an indicator of cell size, while the protein-to-RNA ratio reflects the rate of protein synthesis (20). Therefore, in this study, the cell size and metabolic activity of intestinal cells in the duodenum and jejunum were evaluated by measuring mucosal protein, DNA, and RNA, along with the ratios among these indicators. Assays for these indices were conducted using commercial kits (Nanjing Jiancheng Bioengineering Institute, Nanjing, China) in accordance with the manufacturer’s instructions.

### 2.9 Gene expression analysis

Gene expression for nutrient transporters, intestinal barrier integrity, and inflammatory response was assessed. The primers were designed using online Primer 3 and are shown in Table 2. For real-time quantitative reverse transcription (qRT-PCR) development, total RNA was prepared from the duodenal and jejunal samples using TriZol Reagent (Invitrogen Life Technologies, Carlsbad, CA, USA), and cDNA was synthesized using a cDNA Reverse Transcription Kit (Takara, Dalian, China). qRT-PCR analyses were performed using an ABI 7900 thermocycler (Life Technologies, Carlsbad, CA, USA). The relative mRNA expression of the target genes was normalized to β-actin.

**Table 2:** Primers for quantitative real-time PCR.

Gene	Gene ID	Primer	Sequence (5′–3′)	Size (bp)
*Claudin-1*	NM_001013611.2	Reverse	gtctttggtggcgtgatctt	117
		Forward	tctggtgttaacgggtgtga	
*ZO-1*	XM_015278981.2	Reverse	ggtcagccagatgtggattt	81
		Forward	ccgaagcattccatcttcat	
*Occludin*	NM_205128.1	Reverse	gctgagatggacagcatcaa	97
		Forward	cctctgccacatcctggtat	
*Mucin-2*	NM_001318434.1	Reverse	agtggccatggtttcttgtc	80
		Forward	tgccagcctttttatgctct	
*IL-1β*	XM_015297469.3	Reverse	aggaggtttttgagcccgtca	95
		Forward	gatgtcgaaggactgtgagcg	
*IL-6*	NM_204628.1	Reverse	ctcctcgccaatctgaagtc	100
		Forward	ccctcacggtcttctccata	
*TNF-α*	NM_204267.1	Reverse	agatgggaagggaatgaacc	120
		Forward	actgggcggtcatagaacag	
*IL-10*	NM_001004414.4	Reverse	ctgtcaccgcttcttcacct	86
*CCK*	NM_001001741.2	Reverse	aggttccactgggaggttct	95
		Forward	ccatccagcccatgtagtct	
*FABP-6*	NM_001277700.2	Reverse	caagatcgaaatgggaagga	106
		Forward	attagtcgtggtgcgtcctc	
*SGLT-1*	XM_046928028.1	Reverse	gtgaagacccaggatgccta	92
		Forward	ttcctcttcctccttgctca	
*β-actin*	NM_205518.1	Reverse	gctacagcttcaccaccaca	90
		Forward	tctcctgctcgaaatccagt	
*GAPDH*	NM_204305.1	Reverse	tgggaagcttactggaatgg	88
		Forward	cttggctggtttctccagac	

ZO-1, zonula occludens-1; IL, interleukin; TNF-α, tumor necrosis factor alpha; SGLT-1, sodium-dependent glucose transporter 1; FABP-6, fatty acid-binding protein 6; CCK, cholecystokinin; GAPDH, glyceraldehyde-3-phosphate dehydrogenase.

### 2.10 16S rRNA sequencing and bioinformatics analysis

Isolation of microbial DNA from cecal digesta was performed using a fecal microbial DNA extraction kit (MagBeads FastDNA Kit for Feces, MP Biomedicals, CA, USA). The concentration of DNA was then determined using a Nanodrop 2000 spectrophotometer (Thermo Fisher Scientific, Wilmington, DE, USA), and the purity of DNA was evaluated by 1% agarose gel electrophoresis. The common primer (338F: 5′-ACTCCTACGGGAGGCAGCA-3′ and 806R: 5′-GGACTACHVGGGTWTCTAAT-3′) of region V3–V4 of 16S rRNA genes was used to amplify the bacterial DNA. The amplified products were run on a 2% agarose gel, were purified using a QIAquick Gel Extraction Kit (Qiagen, Germany), and then prepared for sequencing. The library was constructed using the TruSeq Nano DNA LT Library Prep Kit (Illumina, San Diego, CA, USA). The constructed library was quantified using Promega QuantiFluor and qPCR. After the library was validated, the NovaSeq PE250 platform (Illumina, San Diego, CA, USA) was used for sequencing. Paired-end reads from the original DNA fragments were quality-filtered based on specific filtering conditions to obtain high-quality clean tags. The reads were filtered using Quantitative Insights into Microbial Ecology 2 (QIIME2) quality filters. The processed sequences were then subjected to denoising using DADA2 (QIIME2-dada2) to obtain amplicon sequence variants (ASVs). Using the SILVA (release 138) classifier, representative sequences were normalized based on the relative abundance of each sample. Based on the feature ASV table and abundance, alpha and beta diversity, microbial community composition, and differential abundance of taxa were analyzed and visualized in this study.

### 2.11 Statistical analysis

Each pen replicate was used as an experimental unit. The statistical power of at least 0.78 was obtained based on the significance level of 0.05 and the effect size, which was calculated using the mean, replication, and standard deviation using G*Power 3 software (Franz Faul, Christian-Albrechts-Universität Kiel, Kiel, Germany). For the assessment of normality and homogeneity of variances, data were subjected to the Shapiro–Wilk and Levene’s tests, respectively. When normal distribution was confirmed, a two-tailed unpaired *t*-test was performed for comparisons. Data on microbiota were analyzed using R software v3.5.2. Differences in alpha diversity metrics were evaluated similarly. Permutational multivariate analysis of variance was applied to test the effect of treatment on overall community composition on Bray–Curtis distance. Linear discriminant analysis effect size (LEfSe) was used to identify biomarkers that were statistically different between groups based on linear discriminant analysis (LDA) values. The variation was considered statistically significant or as a trend when *p-*value was ≤0.05. Data were presented as mean ± standard deviation.

## 3 Results

### 3.1 Growth performance

As shown in Table 3, growth performance results indicated that supplementing the basal diet with 150 mg/kg β-mannanase significantly reduced both feed intake and the feed-to-weight gain ratio in birds from day 1 to day 21 of age when compared with the Ctrl group (*p* < 0.05). However, no significant difference was observed in body weight at day 21 (*p* > 0.05).

**Table 3:** Effect of dietary β-mannanase on growth performance in broilers at 21 days of age.

Item	Ctrl	β-mannanase
Body weight, g/bird		
1 day	48.66 ± 0.16	48.43 ± 0.14
21 days	848.65 ± 7.89	873.33 ± 9.75
Weight gain (1–21 days), g/bird	799.98 ± 7.95	824.91 ± 9.8
Feed intake (1–21 days), g/bird	1134.88 ± 7.16	1063.35 ± 9.66^*^
Feed to weight gain (1–21 days), g/g	1.423 ± 0.012	1.292 ± 0.006^*^

Data represent means with standard deviation, and values with the letter * are significantly different compared with the control group.

### 3.2 Intestinal growth and morphology

When compared with the Ctrl group, β-mannanase supplementation did not affect the length, weight, and relative weight of the duodenum and jejunum (*p* > 0.05, Table 4). The results of H&E staining also showed that treatment with β-mannanase did not affect the villus height, crypt depth, and their ratio in the duodenum. However, the inclusion of β-mannanase in the diet increased the ratio of villus height to crypt depth in the jejunum, which was accompanied by a numerical increase in villus height and a decrease in crypt depth when compared with the Ctrl birds (*p* < 0.05, Table 5). As far as the mucosal development of intestinal segments is concerned, higher DNA and RNA contents in the duodenum were observed in broilers that received a β-mannanase-supplemented diet than in the Ctrl birds (*p* < 0.05). Similarly, supplementation with β-mannanase in the diet significantly increased the DNA content and protein-to-RNA ratio of the jejunal mucosal homogenate (*p* < 0.05, Table 6).

**Table 4:** Effect of dietary β-mannanase on intestinal length and weight in broilers at 21 days of age.

Item	Ctrl	β-mannanase
Duodenum		
Length, cm	26.525 ± 2.272	27.012 ± 2.550
Weight, g	6.891 ± 1.083	5.841 ± 0.653
Relative weight, g/kg	8.125 ± 1.214	6.887 ± 1.971
Jejunum		
Length, cm	60.925 ± 8.49	64.338 ± 5.901
Weight, g	11.413 ± 1.688	11.311 ± 2.128
Relative weight, g/kg	13.458 ± 1.736	13.338 ± 1.854

Data represent means with standard deviation.

**Table 5:** Effect of dietary β-mannanase on intestinal development in broilers at 21 days of age.

Item	Ctrl	β-mannanase
Duodenum		
Villus height	1410.54 ± 336.59	1428.05 ± 245.59
Crypt depth	155.48 ± 19.89	151.31 ± 31.13
Ratio	9.03 ± 1.58	9.54 ± 1.11
Jejunum		
Villus height	1101.79 ± 199.93	1159.69 ± 197.54
Crypt depth	149.09 ± 23.93	136.48 ± 13.69
Ratio	7.39 ± 0.61	8.47 ± 0.83^*^

Data represent means with standard deviation, and values with the letter * are significantly different compared with the control group.

**Table 6:** Effect of dietary β-mannanase on intestinal development in broilers at 21 days of age.

Item	Ctrl	β-mannanase
Duodenum		
Protein, mg/g	60.80 ± 3.80	62.76 ± 5.24
DNA, mg/g	7.56 ± 1.03	8.70 ± 1.13^*^
RNA, mg/g	0.54 ± 0.04	0.60 ± 0.05^*^
Protein/DNA	8.21 ± 1.46	7.37 ± 1.55
Protein/RNA	113.08 ± 6.36	105.41 ± 11.77
Jejunum		
Protein, mg/g	57.58 ± 4.37	59.57 ± 3.81
DNA, mg/g	7.78 ± 0.84	8.96 ± 0.97^*^
RNA, mg/g	0.59 ± 0.06	0.53 ± 0.09
Protein/DNA	7.51 ± 1.22	6.71 ± 0.81
Protein/RNA	98.48 ± 10.14	115.33 ± 17.7^*^

Data represent means with standard deviation, and values with the letter * are significantly different compared with the control group.

### 3.3 Chyme viscosity of jejunum, digestibility, and nutrient transporters

**Figure 1:**
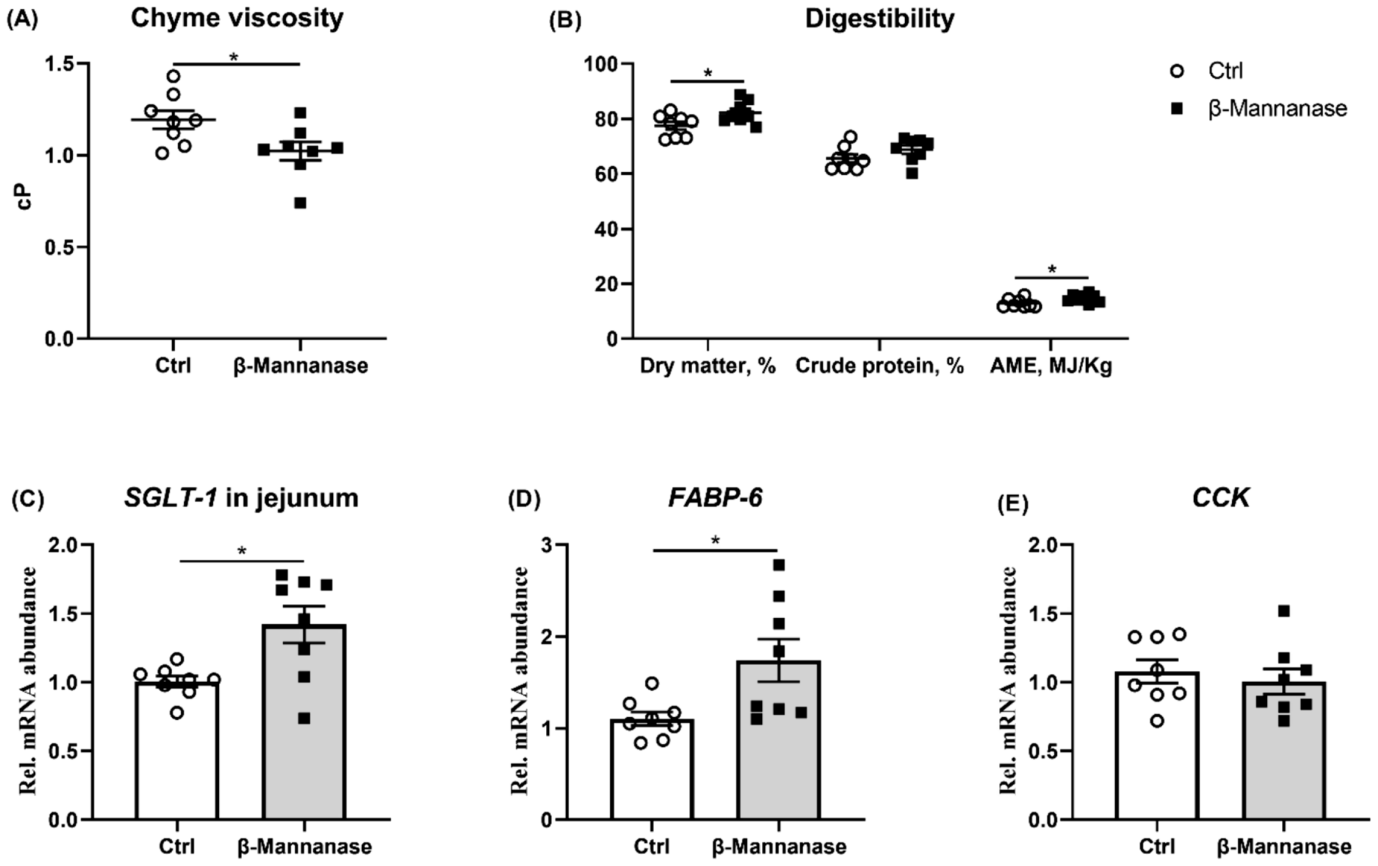
Effect of dietary β-mannanase on chyme viscosity of jejunum **(A)**, nutrient digestibility **(B)**, and the gene expression of nutrient transporters **(C–E)** in broilers at 21 days of age. Data represent means with standard deviation. ^*^Indicates a significant difference between the Ctrl and β-mannanase treatments at a *p*-value of <0.05.

Effects of β-mannanase on digestive characteristics are shown in Figure 1. A diet supplemented with β-mannanase significantly reduced the chyme viscosity of the jejunum of broilers on day 21 (*p* < 0.05, Figure 1A). When compared with the Ctrl group, dietary β-mannanase supplementation increased the digestibility of DM and AMEn (*p* < 0.05), but it did not change the digestibility of crude protein (*p* > 0.05, Figure 1B). The results of nutrient transport showed that the diet with β-mannanase notably upregulated the mRNA expression of sodium-dependent glucose cotransporter 1 (*SGLT-1*) and fatty acid-binding protein 6 (*FABP-6*) as compared with the control diet (*p* < 0.05, Figures 1C,D). However, the expression of cholecystokinin (*CCK*) was similar between the Ctrl and the β-mannanase-fed birds (Figure 1E).

### 3.4 Intestinal barrier integrity

**Figure 2:**
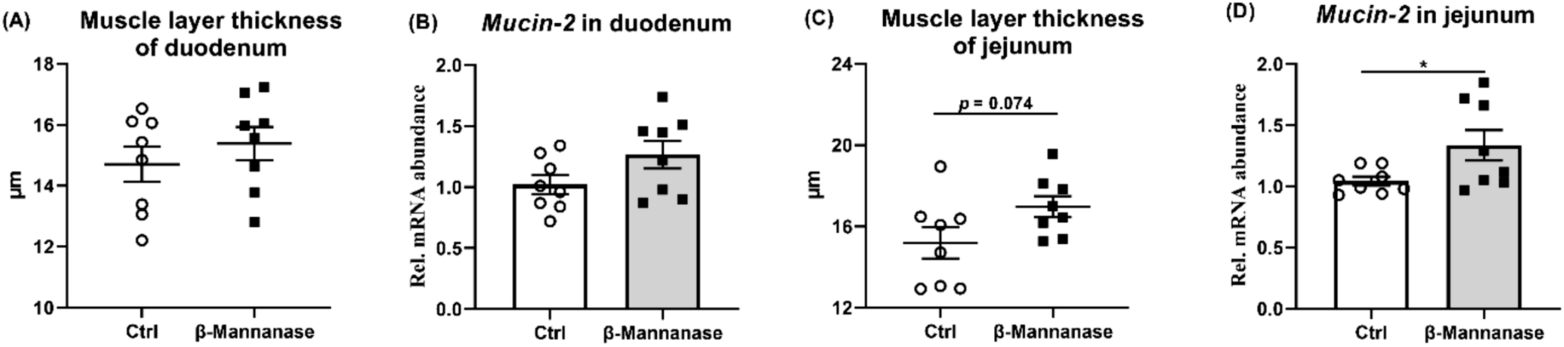
Effect of dietary β-mannanase on the barrier of the mucus layer in the duodenum and jejunum of broilers at 21 days of age. Mucus layer thickness **(A,C)** and the gene expression of mucin-2 **(B,D)**. Data represent means with standard deviation. ^*^Indicates a significant difference between the Ctrl and β-mannanase treatments at a *p-*value of <0.05.

**Figure 3:**
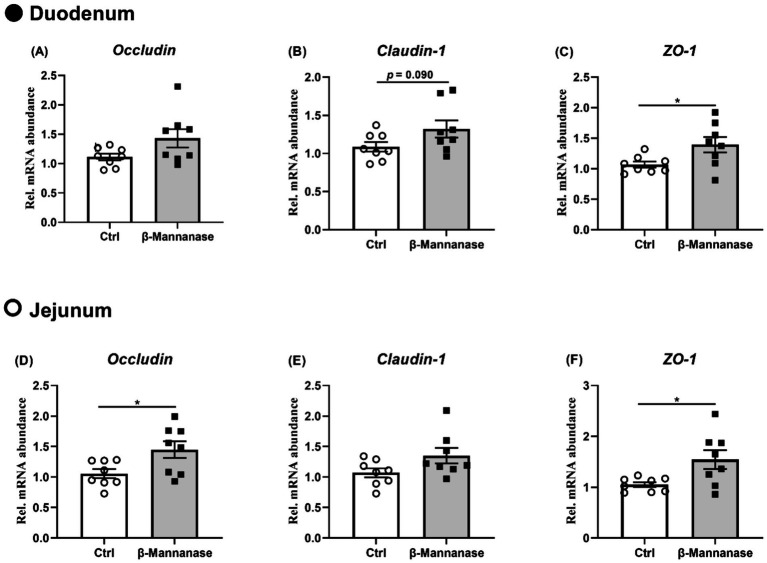
Effect of dietary β-mannanase on the barrier tight junction in the duodenum and jejunum of broilers at 21 days of age. The gene expression included occludin (**A**, **D**), claudin-1 (**B**, **E**), and zonula occludens-1 ZO-1, **C**, **F**) in duodenum and jejunum, respectively. Data represent means with standard deviation. ^*^Indicates a significant difference between the Ctrl and β-mannanase treatments at a *p*-value of <0.05.

**Figure 4:**
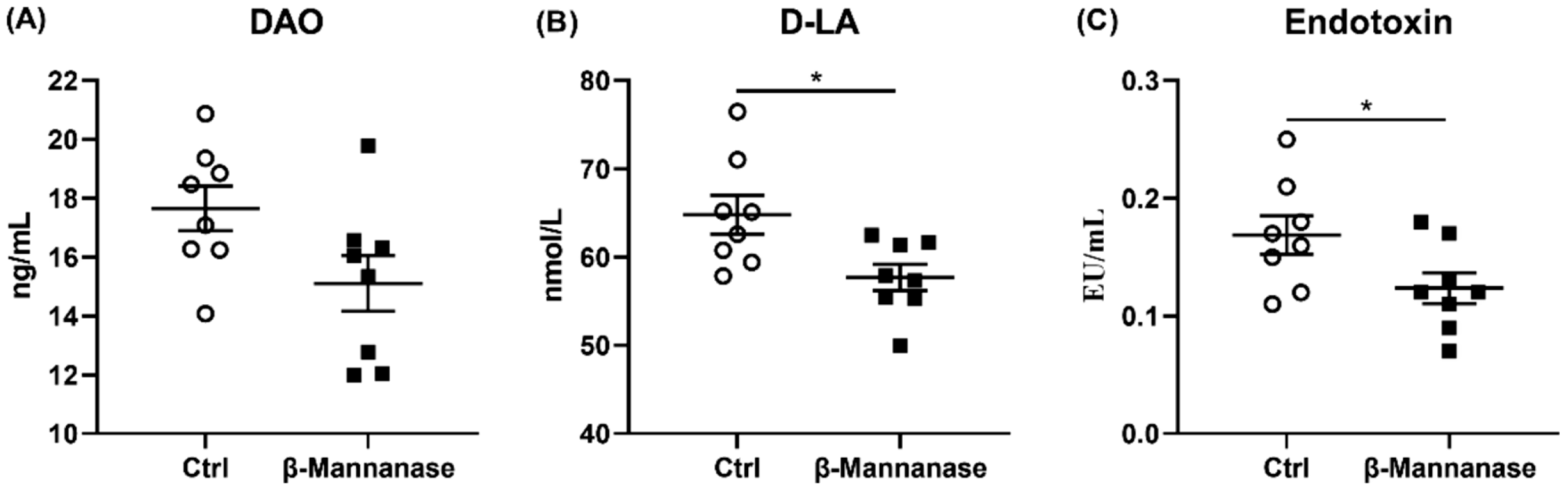
Effect of dietary β-mannanase on intestinal permeability in broilers at 21 days of age. The concentrations of diamine oxidase (DAO) **(A)**, D-lactic acid (D-LA) **(B)**, and endotoxin **(C)** in serum. Data represent means with standard deviation. ^*^Indicates a significant difference between the Ctrl and β-mannanase treatments at a *p*-value of <0.05.

Analysis of the intestinal epithelial barrier via the gene expression of tight junction proteins showed that dietary β-mannanase treatment significantly increased the expression of *ZO-1* in both the duodenum and jejunum, as well as *occludin* mRNA levels in the jejunum (*p* < 0.05). Additionally, there was a tendency toward increased *claudin-1* expression in the duodenum compared with the Ctrl group (*p* = 0.090, Figure 2). Moreover, as shown in Figure 3, the diet containing β-mannanase resulted in no significant changes in mucous layer thickness or *mucin-2* expression in the duodenal mucus (*p* > 0.05), whereas it tended to increase jejunal mucus layer thickness (*p* = 0.074) and notably upregulated *mucin-2* expression (*p* < 0.05). As a result, β-mannanase supplementation decreased (*p* < 0.05) the levels of circulating D-LA and endotoxin, although there was no significant difference in DAO between the two treatments (Figure 4).

### 3.5 Intestinal immune status and inflammatory response

**Figure 5:**
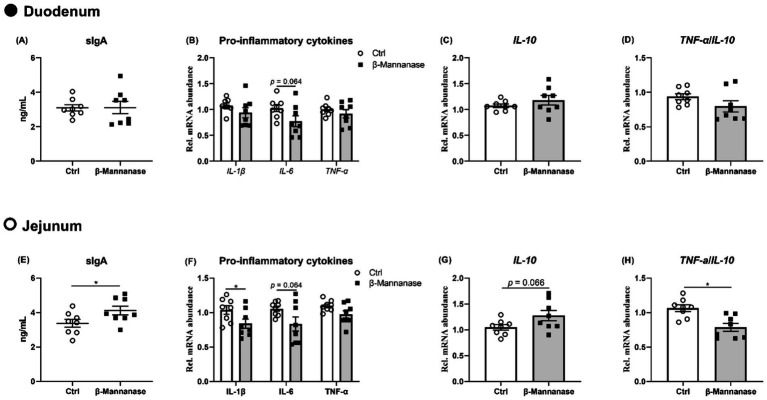
Effect of dietary β-mannanase on inflammatory response in the duodenum and jejunum of broilers at 21 days of age. The concentration of secretory immunoglobulin A (sIgA) **(A,E)**; gene expression of interleukin (IL)-1β, IL-6, tumor necrosis factor (TNF)-α **(B,F)**, and IL-10 **(C,G)**; and the ratio of TNF-α/IL-10 **(D,H)**. Data represent means with standard deviation. ^*^Indicates a significant difference between the Ctrl and β-mannanase treatments at a *p*-value of <0.05.

The influence of dietary β-mannanase supplementation on intestinal immune status and inflammatory response is shown in Figure 5. Experimental treatment did not affect sIgA content or the expression of inflammatory cytokines, although interleukin (IL)-6 mRNA abundance tended to decrease (*p* = 0.064, Figures 5A–D). In the jejunum, β-mannanase supplementation significantly increased sIgA concentration compared with the Ctrl group (*p* < 0.05). Birds fed the β-mannanase-supplemented diet showed reduced expression of the pro-inflammatory cytokines *IL-1β* and *IL-6* while displaying elevated levels of the anti-inflammatory cytokine *IL-10*. This resulted in a lower (*p* < 0.05) tumor necrosis factor-α (*TNF-α*)/IL-10 ratio in the jejunum compared with the Ctrl group (Figure 5H).

### 3.6 Gut microbiota

**Figure 6:**
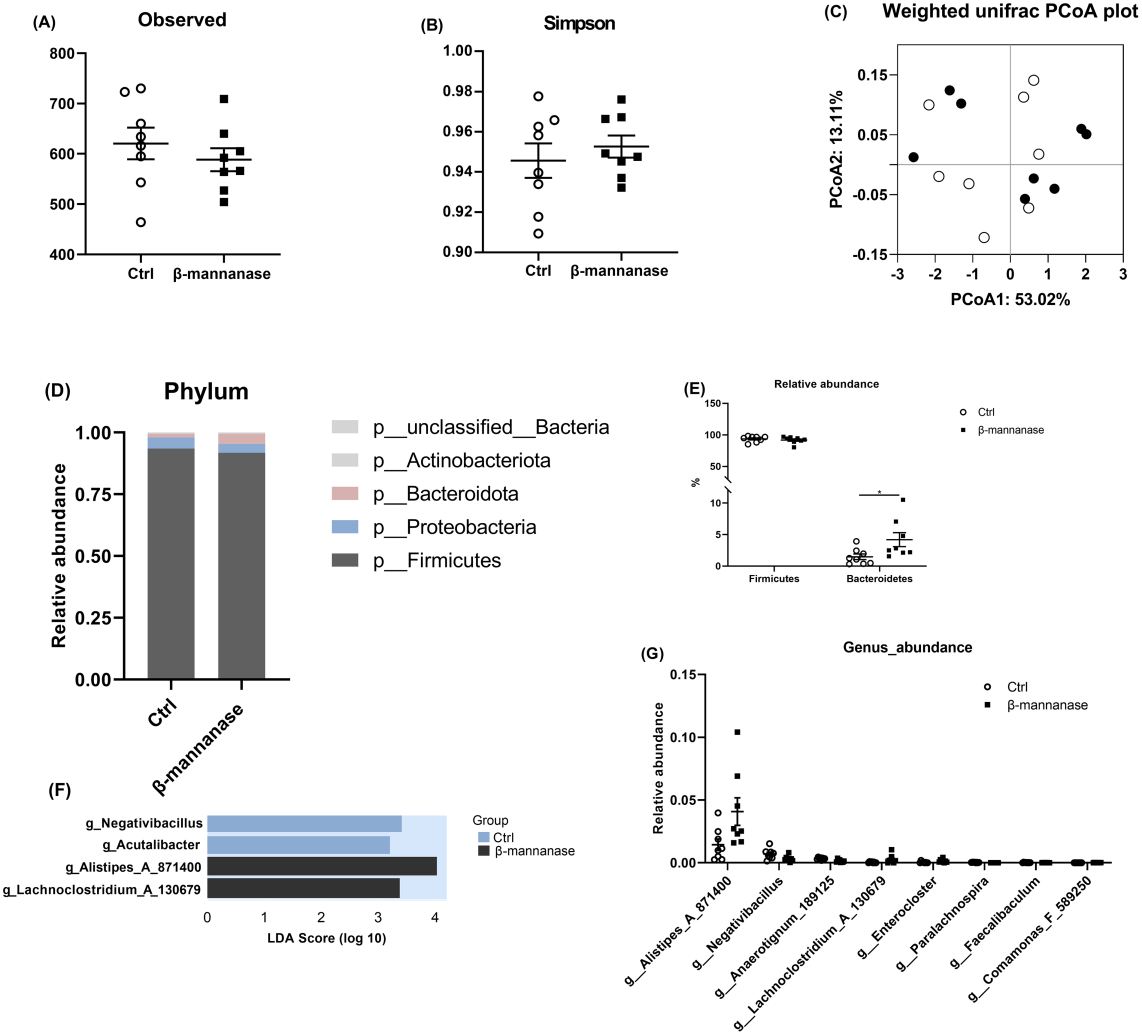
Effect of dietary β-mannanase on gut microbiota in the cecum of broilers at 21 days of age. The observed species and alpha-diversity indexes **(A)**; principal coordinate analysis (PCoA) plots of the weighted UniFrac distance metrics **(B)**; the relative abundance of microbiota at the phylum level **(C-E)**; linear discriminant analysis effect size (LEfSe) test **(F)**; and non-parametric test **(G)** at the genus level.

Concerning the effects of the β-mannanase administration on the cecal microbiota, the components of biodiversity, including species rarity, richness, and evenness, were measured and are shown in Figure 6. On day 21, dietary β-mannanase addition did not affect the observed species count or the Simpson index (*p* > 0.05, Figures 6A,B). Beta (β)-diversity between experimental groups was visualized via principal coordinate analysis (PCoA) based on weighted UniFrac distances, with no significant differences in cecal microbiota β-diversity between β-mannanase-supplemented and Ctrl broilers (Figure 6C). At the phylum level (Figure 6D), Firmicutes and Bacteroidetes were the dominant taxa across all dietary groups. Dietary β-mannanase supplementation significantly increased (*p* < 0.05) the proportion of Bacteroidetes in cecal digesta (Figure 6E). To explore differences between the groups, LefSe analysis showed that *g_Alistipes_A_871400* and *g_Lachnoclostridium_A_130679* were enriched in the cecal digesta of broilers fed the β-mannanase diet (Figure 6F). Moreover, dietary supplementation with β-mannanase significantly increased the relative abundance of *g_Alistipes_A_871400*, *g_Lachnoclostridium_A_130679*, and *g_Enterocloster* (*p* < 0.05), while it lowered the abundance of *g_Negativibacillus*, *g_Anaerotignum_189125*, and *g_Faecalibaculum* (*p* < 0.05, Figure 6G).

## 4 Discussion

Although β-mannanase supplementation in broiler diets has been shown to improve growth performance (17) and nutrient utilization (11) in broilers, the majority of studies have focused on the entire growth cycle. Consequently, limited information is available regarding its effects during the early growth period, particularly on comprehensive intestinal health-related parameters. Therefore, this study was conducted to investigate the effects of dietary β-mannanase supplementation on growth performance, intestinal development, and barrier function in growing broilers fed commercial corn–soybean meal-based diets commonly used in China. These findings indicate that dietary β-mannanase improves gut microecology by reducing chyme viscosity, enhancing intestinal barrier function, and modulating microbiota composition, thereby promoting nutrient utilization and feed efficiency in growing broilers.

The development of villi and microvilli creates a large surface area that is essential for the digestion and absorption of nutrients. In this study, although no change was observed in the overall intestinal size, the addition of β-mannanase improved intestinal morphology by increasing the ratio of villus height to crypt depth in the jejunum. This observation aligns with findings in weaning pigs, where β-mannanase supplementation also increased jejunal villus height and its ratio to crypt depth while reducing crypt depth (16). Furthermore, a recent meta-regression analysis indicated a significant interaction between β-mannanase activity in broiler diets and the villus height-to-crypt depth ratio (11). The improvement in intestinal morphology resulting from β-mannanase intervention may be explained by the fact that dietary supplementation of β-mannanase can prevent intestinal cells from sloughing off and reduce β-mannan-induced immune stimulations in the gastrointestinal tract by breaking down β-mannan, thereby allowing more energy and nutrients to be used for intestinal development (21). In addition, mucosal growth was evaluated by measuring the biochemical parameters of protein, DNA, and RNA, along with the ratios between them. Specifically, changes in DNA reflect variations in cell population, as the DNA concentration in diploid cells is constant. The protein-to-DNA ratio indicates average cell size, while the protein-to-RNA ratio reflects the rate of protein synthesis (20). In the present study, higher DNA and RNA concentrations, along with the increased ratio of protein to RNA in the duodenal and jejunal mucosa, suggest that dietary β-mannanase promotes intestinal development in early-stage broiler chickens by enhancing cell proliferation and protein synthesis.

Functional improvements are likely underpinned by an enhanced intestinal environment and positive morphological changes in the small intestine. β-mannanase enhances nutrient utilization by counteracting the anti-nutritional effects of β-mannans, which are pervasive in poultry feed (3, 4). In this study, β-mannanase treatment upregulated the mRNA levels of the nutrient transporters SGLT1 and FABP6 in the jejunum. SGLT1 mediates the apical absorption of monosaccharides such as glucose and galactose by coupling their transport to the Na+ gradient generated by Na^+^–K^+^-ATPase (22). FABP6, conversely, is critical for the cellular uptake and transport of lipids and bile acids (23). The coordinated upregulation of these genes suggests that β-mannanase improved nutrient utilization by simultaneously enhancing the absorption capacity for both carbohydrates and lipids. Moreover, highly viscous digesta can impede the diffusion and convective transport of digestive enzymes, thereby reducing their contact with substrates (24). In the current study, dietary β-mannanase supplementation significantly reduced jejunal chyme viscosity in 21-day-old broilers. This reduction likely enhanced the activity and efficiency of digestive enzymes by improving their access to substrates, which explains the concurrent increases in dry matter and metabolizable energy digestibility. These improvements in digestion were consistent with the observed upregulation of nutrient transporters. As a positive consequence, supplementing diets with 150 mg/kg β-mannanase improved broiler performance during the starter stage, as evidenced by a reduced feed-to-weight gain ratio and a numerical increase in body weight at day 21. This finding was supported by a meta-analysis confirming that β-mannanase supplementation positively affects the feed conversion ratio in broilers (3). Collectively, these findings demonstrate that dietary β-mannanase improved nutrient utilization and feed efficiency in growing broilers by reducing chyme viscosity and promoting intestinal development.

In addition to selectively absorbing nutrients, the intestinal tract plays a key role in confronting foreign substances by neutralizing or eliminating toxins and enteric pathogens (25). Several defense mechanisms are provided by the epithelium of the intestinal tract, one of which is the establishment of a selective permeability barrier (26). DAO, D-LA, and endotoxin are convenient and accessible biomarkers whose levels in serum can reflect intestinal barrier permeability (27). In this experiment, the dietary supplementation of β-mannanase to the diet reduced the levels of D-LA and endotoxin in serum, thereby improving the permeability of the intestinal epithelium. Similar to these results, the manipulation of β-mannanase in the feed alleviated the increase in intestinal permeability caused by lower energy levels and reduced the serum DAO and endotoxin levels (12). Additionally, tight junctions of the intestinal epithelium, the multi-protein complexes, are the basis for barrier permeability, and the functioning of the involved proteins is important for barrier function. Among numerous tight junction proteins, Occludin, Claudin-1, and ZO-1 are the most representative. Occludin and Claudin-1 are transmembrane proteins that form the intestinal barrier, while ZO-1 is the junction protein between transmembrane proteins and the cytoskeleton, and their interaction is crucial to tight junction assembly and maintenance (26). The results from this study showed that the gene expression of *occludin* and *ZO-1* in the jejunum has been upregulated after birds were fed a β-mannanase-supplemented diet, implicating a promoting impact of β-mannanase on intestinal barrier function in broilers. In addition to the mechanical barrier, the mucus layer is also an integral component of host defense against pathogen infection of the intestinal epithelium. Intestinal mucus forms an outer barrier overlying the epithelium, which is produced by goblet cells and forms a highly organized glycoprotein network, primarily consisting of mucin-2 (28, 29). By assessing the intestinal thickness of the mucus layer directly and the gene expression of *mucin-2* indirectly, broilers fed a diet with β-mannanase were found to enhance the mucus barrier integrity in the present trial, which was reflected by a slightly thickened mucus layer as well as a significantly upregulated *mucin-2* gene expression in the jejunum. These observations corroborate previous research that identified alterations in gut functional pathways in the jejunum of β-mannanase-fed birds (30). These data highlight the beneficial role of β-mannanase in preserving intestinal barrier integrity.

The intestine is specially adapted to colonization by commensal bacteria, which form a multi-layered microbial barrier that aids in digestion and nutrition, host defense, and ultimately regulating the immune function (26). In general, the distribution and structure of gut microbes are relatively stable, and the microbial composition is relatively balanced (31). A previous study has shown that β-mannans in feed can lead to microbial disturbances in laying hens and that the addition of β-mannanase can mitigate this adverse effect (32). Although dietary β-mannanase supplementation did not affect the alpha-diversity of microbial species in the cecum of 21-day broilers, a noticeable change in the microbial communities was observed in the current study. Consistent with the increased proportion of the phylum Bacteroidetes, the genus *Alistipes* within this phylum was also found to be significantly increased with β-mannanase addition. In addition, genus *Lachnoclostridium,* which belongs to the family *Lachnospiraceae*, was simultaneously observed to be enriched in broilers fed the β-mannanase-supplemented diet. These results from our study are in agreement with a previous study of broiler chickens fed with low-energy diets with or without β-mannanase, and it was reported that the addition of β-mannanase increased the average abundances of *Alistipes* and *Lachnospiraceae_unclassified* in the cecum of broilers at 42 days (33). The bacteria of the genus *Alistipes* and members of *Lachnospiraceae* are mostly found in healthy human gastrointestinal tract microbiota and are identified as being involved in carbohydrate catabolism (34, 35). Indeed, diet and host carbohydrates have confirmed to shape the gut microbiota by providing a major nutrient source for gut-dwelling microbes. This may explain why alterations in carbohydrate metabolism due to β-mannanase supplementation promoted the enrichment of *Lachnospiraceae* in the current study. Additionally, *Enterocloster* has recently been identified as a protective species against *Salmonella typhimurium* infection via selective upregulation of resistin-like molecule β and cell cycle pathways in cecal epithelium, coupled with an increase in mucosal T-regulatory cells, which, in turn, mitigates infection-induced pathology (36). An increase in the relative abundance of *Enterocloster* due to the use of β-mannanase might imply that dietary β-mannanase exerts a positive role in cecal microbiota composition. Taken together, the current findings indicate that β-mannanase could contribute to intestinal health in broilers at an early stage by shaping a beneficial structure of cecal microbiota.

Both the intestinal barrier integrity and microbiota composition are closely tied to both immune status and inflammatory responses. Intestinal epithelial cells maintain immunoregulatory function by coordinating immune responses, a process that can be disrupted when β-mannans present in the feed are recognized by innate immune cells, potentially triggering immune activation (37, 38). In this regard, β-mannanase supplementation has been reported to modulate the immune response of broiler chickens because of the elimination of β-mannans-induced immune-related signaling within the jejunum (30) and the elevated production of immunoglobulins (Ig), either in serum (21) or in the intestine (39, 40). Similarly, the capability of β-mannanase in immunoregulation was verified in the current study by the increased sIgA in the jejunum. This effect could be attributed to mannooligosaccharides (MOS) generated by β-mannanase hydrolysis. MOS function as immunostimulants by occupying mannose receptors while also being recognized as pathogen-associated molecular patterns, thereby collectively stimulating the gut-associated lymphoid tissue (41, 42). While these mechanisms were not directly determined, they provide a potential explanation for the observed improvements in the immune status of broilers in the early stage. Alternatively, the intestinal immunity is significantly associated with inflammatory response. Evidence of a role for β-mannanase in the protection against intestinal inflammation has emerged from previous studies of broiler chickens and pigs on either reduced protein or energy diets (12, 43, 44). The results of this study showed that broilers fed the diet with β-mannanase supplementation had reduced jejunal *IL-1β* and *IL-6* transcription. These two major pro-inflammatory cytokines are mainly secreted by monocytes and macrophages, which can initiate the host inflammatory response and promote the removal of infectious agents (45, 46). Despite the unaltered transcriptional level of the other pro-inflammatory cytokine TNF-α, which is known to promote early immune responses and acute phase reactions (47), the TNF-α-to-IL-10 ratio, as a marker of the balance between a pro-inflammatory and anti-inflammatory state, was reduced in birds fed diets supplemented with β-mannanase, indicating ameliorated jejunal inflammation. This anti-inflammatory effect was accompanied by a higher jejunal *IL-10* transcriptional level. The elevated IL-10 is functionally significant, as this critical anti-inflammatory cytokine inhibits excessive immune activation and contributes to the maintenance of intestinal barrier integrity (48). As a result, it is possible that the addition of β-mannanase is conducive to maintaining overall immune homeostasis and preventing excessive inflammatory responses.

## 5 Conclusion

A major limitation of this study lies in its experimental design, which only included a basal diet and a basal diet supplemented with 150 mg/kg β-mannanase. Further research incorporating low-energy diets and multiple dosages of this enzyme is therefore warranted. Within these limitations, the results of this study suggest that dietary supplementation with 150 mg/kg β-mannanase can improve nutrient utilization and feed efficiency in growing broilers. These beneficial effects are likely associated with reduced chyme viscosity, strengthened intestinal barrier function, and an optimized gut microbiota composition.

## Data Availability

The data presented in the study are deposited in the NCBIdatabase accession number PRJNA1467427.
